# Properties and Strength Prediction Modeling of Green Mortar with Brick Powder Subjected to a Short-Term Thermal Shock at Elevated Temperatures

**DOI:** 10.3390/ma14216331

**Published:** 2021-10-23

**Authors:** Maciej Szeląg, Joanna Styczeń, Roman Fediuk, Renata Polak

**Affiliations:** 1 Department of Construction, Lublin University of Technology, Nadbystrzycka 40, 20-618 Lublin, Poland; maciej.szelag@pollub.pl; 2 Polytechnic Institute, Far Eastern Federal University, 690922 Vladivostok, Russia; fedyuk.rs@dvfu.ru; 3 Department of Thermal Technology and Food Process Engineering, University of Life Sciences in Lublin, Głęboka 31, 20-612 Lublin, Poland; renata.polak@up.lublin.pl

**Keywords:** brick powder, intelligent modeling, mechanical properties, mortar

## Abstract

The cement industry is responsible for 8% of global CO_2_ production. Therefore, a clear trend has been observed recently to replace to some extent the main binder of cement composites with environmentally friendly or recycled materials with a lower carbon footprint. This paper presents the effect of brick powder (BP) on the physico-chemical and mechanical properties of cement mortars. The effect of a short-term thermal shock on morphology and strength properties of green mortars was investigated. BP addition caused increase in porosity and decrease in compressive and flexural strength of mortars. The best results were obtained for samples with 5% wt. BP addition. Above this addition the strength decreased. The mechanical performance of the samples subjected to thermal loading increased compared to the reference samples, which is the result of a process called as the “internal autoclaving”. The BP addition positively affects the linear shrinkage, leading to its reduction. The lowest linear shrinkage value was achieved by the mortar with the highest BP addition. An intelligent modeling approach for the prediction of strength characteristics, depending on the ultrasonic pulse velocity (UPV) is also presented. To solve the model problem, a supervised machine-learning algorithm in the form of an SVM (support vector machines) regression approach was implemented in this paper. The results indicate that BP can be used as a cement replacement in cement mortars in limited amounts. The amount of the additive should be moderate and tuned to the features that mortars should have.

## 1. Introduction

Globally, cement production has a negative impact on the environment [1,2,3]. This is related to the use of a huge amount of geological natural resources in the form of aggregates, and the fact of high energy consumption in the clinker firing process in cement production [4,5]. As a result, the cement industry accounts for 7–8% of global CO_2_ emission [6,7] and 12–15% of global industrial energy consumption [8,9]. Simply improving the cement manufacturing process is not enough, so it is important for the environment to try to reduce its production at the expense of replacing it with secondary materials. An effective solution is the use of pozzolanic materials, which can partially replace the Portland clinker and thus reduce energy consumption, pollutant release and give cement composites the desired characteristics [10].

Pozzolanic additives modify the phase composition of the cement matrix, causing a reduction in the amount of portlandite and the CaO/SiO_2_ ratio in the C-S-H phase. The main cause of Ca(OH)_2_ reduction in hardened cement paste is the formation of silica-rich hydrogranates (CxAySmHn) and hydrogelenite (C_2_ASH_8_) [11]. The presence of pozzolans among the reactants causes a number of changes: a decrease in the hydration heat, an increase in the content of the C-S-H phase, a decrease in the content of calcium hydroxide, changes in the water demand and workability of mortars and concretes, as well as an increase in their strength over a longer maturation period [12].

One of the additives for cement composites can be the brick powder (BP) which is a construction and demolition waste (CDW). The use of construction wastes in the form of crushed or ground bricks can reduce the environmental costs that countries have to pay for depositing them [13]. In China, waste clay bricks are a major component of construction wastes, and the recycling rate of CDW is less than 10% [14,15]. The CDW is often moved to rural and suburban areas where it is deposited in the open space or buried underground. This leads to high disposal fees and severe secondary environmental pollution [16,17]. In the European Union, CDW production reached 868 million tons in 2014, accounting for 35% of all wastes generated by the EU [18]. The main components of CDW are concrete and brick. This wastes mostly comes from construction or demolition of buildings, renovation activities and natural disasters [19,20]. The EU Waste Framework Directive (2008/98/EC) mandates 70% recycling of construction waste [21]. According to the researchers’ reports, wastes from fired clay brick generally exhibits some pozzolanic activity (reacts with calcium hydroxide) [22,23,24,25,26]. This activity is related to its degree of fineness, among other factors. Ma et al. [27] in their study showed that the specific surface area of BP is fundamental on its calcium binding ability. When the fineness of BP was higher than that of cement, the compressive strength (f_c_) was 15% higher compared to a reference mortar. The f_c_ decreased when its fineness was close to or lower than that of cement.

In recent years, the use of BP as a partial replacement for Portland cement in cement composites has received much attention [28,29,30,31,32]. Research results suggest that when BP replaces cement at no more than 30%, the strength of mortar or concrete is similar to reference samples. Researchers from the team of Xiong et al. [33] compared the properties of cement composites with the addition of fly ash and BP. The researchers showed that the 7-day BP activity rate was the same as for fly ash, while the 28-day BP activity rate was 14.3% higher. The flexural and compressive strength of cement mortar decreased with the increase of BP and fly ash as a cement substitute. Moreover, at the same amount of cement substitution, the 28-day compressive strength of mortar with BP was higher than that of mortar with fly ash. With the addition of BP from 0% to 50% as a cement substitute, the fluidity of the mortar first increased and then decreased.

Zhao et al. [34] studied cement composites in which 30% of cement was replaced by a slag and BP. The results show that 30% of slag and BP can increase the hydration value of C_3_S by 11.4~13.7%, when the hydration of C_3_A is more affected by slag. The cumulative heat of hydration after 144 h was reduced by 4.1~21.3% compared to pastes with the Portland cement alone. The BP improves the mechanical strength of mortars before and after 90 days of maturation, respectively. During the first 90 days of hydration, the use of blast furnace slag in the mortars resulted in a decrease in the amount of portlandite compared to mortars with BP, while after this period the amount of portlandite was less in the BP mortars. After 90 days, mortars with BP exhibited higher strength than mortars with blast furnace slag. Similar positive strength characteristics were noted by other researchers using ceramic waste as a concrete additive [35,36,37]. The use of BP improves the long-term strength of concrete. Researchers have shown that the compressive strength of concrete containing 10% BP is 32% higher than that of plain concrete after a long curing time [38]. The use of ceramic waste both as cement replacement and fine aggregates significantly improved the compressive strength, durability, and resistance of mortar to sulfate corrosion [39].

Li [40] studying the pozzolanic activity of ceramic wastes showed that they are rich in silica (SiO_2_) and alumina (Al_2_O_3_) which affects the high pozzolanic activity. Schackow et al. [41] investigated the BP effect on the strength and ion exchangeability of mortars. The researchers observed that the BP addition can increase the strength of cement mortar to some extent, but it can decrease the resistance to the negative effects of chloride ions and sulfuric acid. Vejmelkova et al. [42] investigated the mechanical properties of high performance concrete (HPC) in which Portland cement was replaced with different amounts of finely ground ceramic waste—up to 60%. Mechanical properties and water permeability were not significantly affected when BP was used up to 20%, while salt crystallization pressure resistance was only satisfactory up to 10% of the BP addition, and chemical resistance (to Na_2_SO_4_ and MgCl_2_) was retained up to 40%. Ortega et al. [43] analyzed the properties of mortars after long time that contained up to 20% BP as a cement replacement. Mortars with 10% and 20% BP addition, due to the modification of the air pore structure, showed good performance properties over a long observation period (400 days).

Considering the positive effects of BP on the performance of cement composites that other researchers have obtained in their studies, the authors undertook research on its influence on the properties of cement mortars. To date, researchers have mainly focused on the use of BP in concretes and its effect on mechanical properties. The authors of this paper focus on the BP effect on physico-chemical and mechanical properties, and the effect of thermal shock on the morphology and strength properties of mortars. Mortars with BP at 5%, 10%, 15% and 20% content and reference samples without BP were tested. The raw materials were analyzed by XRD and XRF methods. Additionally, the authors made prediction modeling of the strength properties of the BP mortars before and after the thermal load using an intelligent modeling approach.

## 2. Materials and Methods

### 2.1. Materials

Five cement mortar recipes were prepared with different BP amounts. Each mixture was prepared with a cementitious binder base. The resulting recipes differed in the amount of BP addition, which was 5%, 10%, 15% and 20%, respectively, replacing the weight of cement. A comparison mortar was also prepared. The w/b (water/binder) ratio was 0.5. The compositions of the mortars are shown in Table 1.

**Figure 1:**
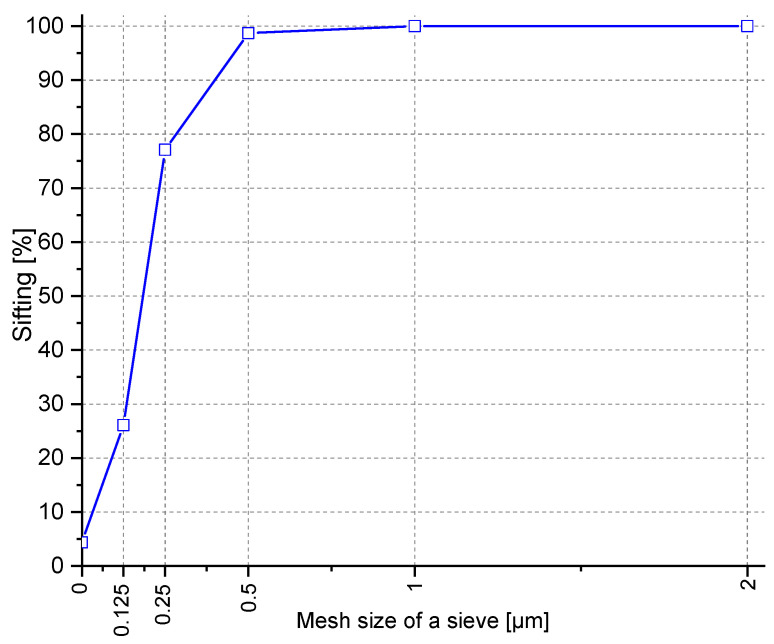
Particle size distribution curve of the quartz sand.

The following components were used in the mortars:

- ordinary Portland cement (OPC) CEM I 42.5R with the following properties: beginning of setting time—185 min, end of the setting time end—240 min, compressive strength after 2 days—30.5 MPa, after 28 days—59.4 MPa, volume change—0.9%, insoluble residue—0.64%, loss of ignition—3.22%.
- brick powder (BP) obtained from a milled brick waste with a 0–2 mm fraction.
- quartz sand (0–2 mm) dominated by SiO_2_—95.3%, specific gravity—2650 kg/m^3^, water absorption—1.2%. The analyzed quartz sand is classified as a fine aggregate (Figure 1). The 0.25–0.5 fraction dominates, with a content of 51%. The content of grains with a diameter larger than 0.5 mm is 21.8%. According to these values, the aggregate used for cement mortar testing is a medium-grained sand.

### 2.2. Methods

A set of rectangular specimens with dimensions of 40 × 40 × 160 mm^3^ and 100 × 100 × 100 mm^3^ were prepared according to the PN-EN 196-1:2016-07 [44]. The mixture was placed in steel moulds (GEOLAB, Warsaw, Poland) in two layers, successively compacted on a vibrating table. After 24 h, the specimens were removed from the molds and then placed in a climatic chamber for 28 days at an average temperature of 20 °C and average relative humidity of 50%.

Temperature loading consisted of preheating the furnace (CZYLOK, Jastrzębie-Zdrój, Poland) to 250 °C and then placing the specimens in it. Placing the mortars directly in the preheated furnace corresponds to the thermal shock loading. The residence time of the samples in the furnace was 4 h. After this time, the specimens were taken out and left at the room temperature for natural temperature drop. 

The flexural strength test (f_cf_) was conducted on three specimens according to the EN 12390-5 [45]. The compressive strength test (f_c_) was carried out according to the EN 12390-3 [46,47]. The result is the average of six specimens formed after the f_cf_ test. 

The particle size distribution was determined using a Mastersizer 3000 (Malvern Panalytical, Malvern, UK) with HYDRO EV (Malvern Panalytical, Malvern, UK) overlay. Isopropanol was used as the dispersing medium. 

The X-ray diffraction (XRD) analysis was used to determine the mineral composition of the raw materials. Tests were carried out on powdered samples, ground in an agate mortar. The X’pertPRO MPD diffractometer (Panalytical, Almelo, The Netherlands) with PW 3020 goniometer (Panalytical, Malvern, UK), Cu copper lamp (CuKα = 1.54178 Å) (Panalytical, Malvern, UK) and graphite monochromator (Panalytical, Malvern, UK) were used for this purpose. The analysis was performed over an angular range of 5–65° 2θ, with a measurement step size of 0.02° 2θ and a time of five seconds per step. The results obtained were analyzed using the X’Pert Highscore software.

The chemical composition of BP and cement was investigated using the X-ray fluorescence method (XRF). An Epsilon 3 spectrometer (Panalytical, Almelo, The Netherlands) with a Rh 9 W, 50 kV, 1 mA X-ray tube, a 4096 channel spectrum analyzer, six measurement filters (Cu-300, Cu-500, Al.-50, Al.-200, Ti, Ag), and a high-resolution solid-state SDD detector cooled by a Peltier cell were used for the study. Prior to testing, the samples were dried to a solid mass, grinded in an agate mortar, and then placed in plastic cups approximately 5–8 g.

The apparent density of hardened mortars (D) was tested according to the PN-EN 1015-10 [48] after 28 days of maturation. The result given in the paper is the arithmetic mean from three samples. The specific density (ρ) was tested using pycnometers—the average of three samples. Tightness (T) was calculated as the ratio of ρ and D—arithmetic mean of three samples. The total porosity (P_T_) determines how much of the total volume of the material is a pore volume—arithmetic mean of three samples.

A linear shrinkage (S) test was performed using a Graf-Kaufman apparatus (EMEL, Warsaw, Poland). Measurements were taken after 3, 7, 14, 21, and 28 days of maturation—arithmetic mean of 3 samples. The samples for this study were matured in a desiccator. 

A volume water absorption (W_A_) was determined as the ratio of the difference of the weight of water-soaked and dry material to the volume of the sample—arithmetic mean of three samples.

The ultrasonic pulse velocity (UPV) test was conducted on 100 × 100 × 100 mm cubic samples. The specimens were tested after 28 days of maturation (reference specimens) and specimens after the thermal loading. The Pundit Lab with 54 kHz UPV transducers (Proceq, Schwerzenbach, Switzerland) was used for the test. The result presented in this paper is the arithmetic mean of the three measurements. This test was performed based on the ASTM C 597 [49] in direct transmission mode. The test resulted in the ultrasonic pulse velocity through the tested mortars. 

The morphology was examined using a Quanta 250 Scanning Electron Microscope (FEI, Hillsboro, OR, USA). The apparatus is equipped with an electron gun with a LaB6 cathode and a system that allows the examination of chemical composition. The examination was carried out in the so-called “high vacuum” mode of operation, in the secondary electron light at an accelerating voltage in the range of 10–15 keV. The samples were immersed in isopropanol prior to testing to stop the hydration process, then dried and sputtered with a conductive carbon layer.

## 3. Results and Discussion

### 3.1. Ordinary Portland Cement (OPC)—CEM I 42.5 R 

**Figure 2:**
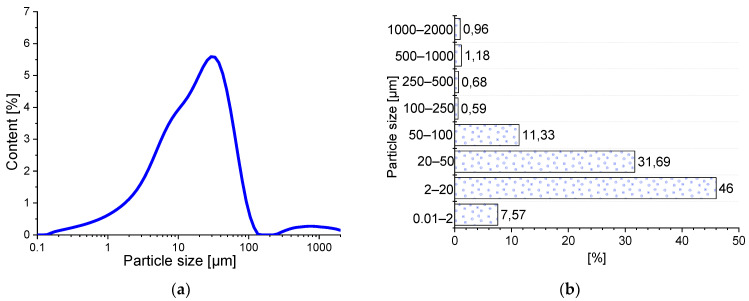
Particle size distribution of the CEM I 42.5 R: (**a**) the graining curve; (**b**) percentage of grains in different size classes.

The specific density of the cement was 2.93 g/cm^3^ and the specific surface area tested by the Blaine apparatus was 4139 cm^2^/g, respectively. Both tested values were higher compared to BP, and the difference was 8.9% and 19.4%, respectively. The results of the cement particle size distribution showed a mono-modal character (Figure 2). The material is dominated by grains with particle size in the range of 2–50.0 µm, accounting for 89.0% of the total grains. The presence of grains with sizes in the range of 200–1000 µm may indicate a slight clumping of the binder grains.

**Figure 3:**
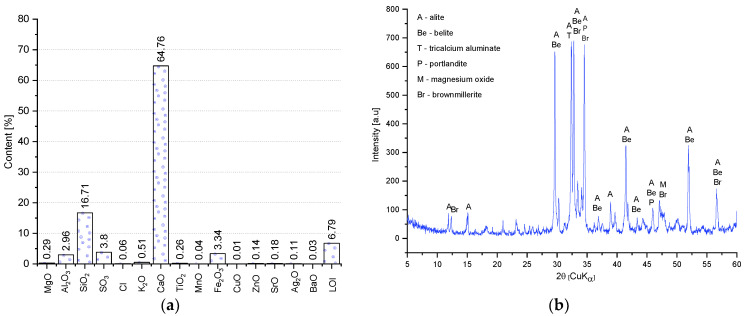
Chemical characteristics of the CEM I 42.5 R: (**a**) chemical composition; (**b**) X-ray diffraction (XRD) pattern.

The chemical composition (by means of the XRF) of the CEM I 42.5R and the XRD pattern of the mineral composition of the cement used are shown in Figure 3. The dominant components of the cement is CaO, which accounts for 64.7%, and SiO_2_ (16.7%). Trace amounts of elements were also detected in the cement: Cl, MnO, CuO, ZnO, SrO, Ag_2_O, and BaO with a total content of 0.57%. Due to their trace amounts, they have a marginal effect on the physical and chemical properties of the binder. The XRD pattern shows all the major phases that have been identified by their characteristic interplanar distances d_hkl_. The cement used is predominantly alite (d_hkl_ = 2.78; 2.76; 3.05; 2.61; 2.74 Å), belite (d_hkl_ = 2.78; 2.79; 2.75; 2.62; 2.72 Å), tricalcium aluminate (d_hkl_ = 7.61 Å), and brownmillerite (d_hkl_ = 7.12, 7.45 Å).

### 3.2. BP—Brick Powder

The BP specific density was 2.67 g/cm^3^ and the specific surface area tested by the Blaine apparatus was 3337 cm^2^/g. This surface area is 19.4% less than the specific surface area of the cement. The pozzolanic activity of BP is related to particle size and specific surface area. An increase in the fineness of the grains leads to an increase in pozzolanic activity, but too much fineness can lead to the BP agglomeration and thus to a deterioration of the mixture properties. For the finest BP particles, their higher specific surface area leads to reduced workability and increased water demand. Grellieri et al. [50] studied mortars with BPs with different specific surface areas. Mixtures with BP of the specific surface area similar to that used in the present study obtained the best f_c_ results among the other mortars after 90 days.

**Figure 4:**
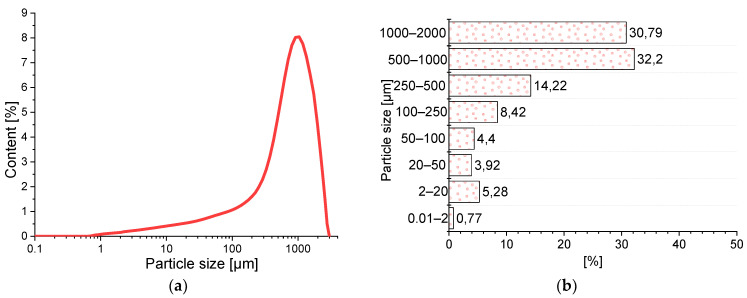
Particle size distribution of the brick powder (BP): (**a**) the graining curve; (**b**) percentage of grains in different size classes.

The results of the BP particle size distribution (Figure 4) show an approximately mono-modal character. The material is dominated by grains with particle sizes in the range of 250–1000 µm, which account for 76.1% content. There are also much smaller grains in the range of 0.2–20 µm (5.3%). The grain size characteristics indicate that the applied BP is closer in grain size to finely ground sand than to binder. Thus, the pozzolanic activity is very low and the role of BP in the mortars tested will be mainly that of a microfiller.

**Figure 5:**
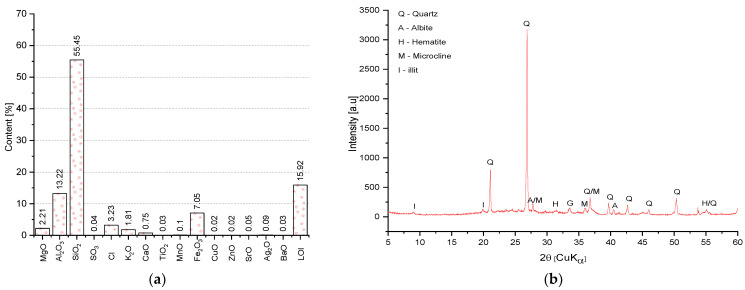
Chemical characteristics of the BP: (**a**) chemical composition; (**b**) XRD pattern.

The chemical composition (by means of the XRF) and inorganic crystalline phases identified on the XRD pattern are shown in Figure 5. The main constituent of BP is SiO_2_, which accounts for (55.5%), Al_2_O_3_ (13.2%), and Fe_2_O_3_ (7.1%). In the chemical composition of BP also can be found trace amounts of elements that do not affect the physical and chemical properties of BP. These include: Cl, MnO, CuO, ZnO, SrO, Ag_2_O, BaO. The total content of these elements is 1.06%. 

The results of the mineral composition test indicate the presence of quartz in large amounts, which is the main silica phase (sand used to adjust the plasticity of the brick mixture). Also detected were iron oxide (hematite—used to reduce the firing temperature and favor the formation of liquid phases), illite (the main mineral component of the clay used to make bricks), potassium and sodium feldspar (microcline, albite).

### 3.3. Hardened Properties of Cement Mortars with BP 

#### 3.3.1. Physical Properties

The physical properties of the mortars are shown in Table 2. The addition of BP decreased the apparent density of the cement mortars. The P5, P10, P15, P20 series had lower apparent density than the classic P0 mortar. With the addition of BP, the specific density also decreased. This is due to the replacement of cement by BP which has a lower specific density by 8.9% compared to the CEM I 42.5R. This translates into an average decrease of 7.4% for all samples. The largest decrease was recorded for P20 and amounted to 14.5%.

The BP addition resulted in a decrease in tightness relative to the P0 mortar. The highest tightness was achieved for P0 samples (81.2%). The decrease in the tightness is due to the increase in porosity of the samples with BP. Samples P20 (27.8%) had the highest total porosity. The increased porosity is directly related to the structure of BP, which is characterized by an extensive capillary pore system. Similar results were noted by other researchers who showed that the BP incorporation would produce a higher microstructure refinement, highlighting the greater proportion of pores with diameters in the range < 10 nm [51]. The percentage of Hg retained at the end of the porosimetry test was higher for mortars with BP. This parameter provides qualitative information related to the pores’ tortuosity [52]. Therefore, the results of Hg retained would indicate a greater pore network tortuosity for samples with BP and suggesting a higher pore refinement produced by this addition. The increase in porosity may also indicate a decrease in the density of the microstructure resulting in a decrease in the amount of calcium silicate hydrates from the pozzolanic reaction [52,53].

The comparative P0 mortar was characterized by the lowest water absorption among the tested samples. This parameter increased with the addition of BP. The water absorption of P20 mortar was 6.8% higher than that of the P0 mortar. Mortars with the highest total porosity achieved the highest W_A_. Similar relationships were observed by Giosuè et al. [54]. The researchers found that the initial (linear) phase of capillary water absorption corresponds to the filling of capillary pores with larger sizes, while the second phase corresponds to the filling of smaller pores [55,56]. Given that the test was applied over a short period of time (90 min), large pores have a greater effect on capillary water absorption than small pores.

**Figure 6:**
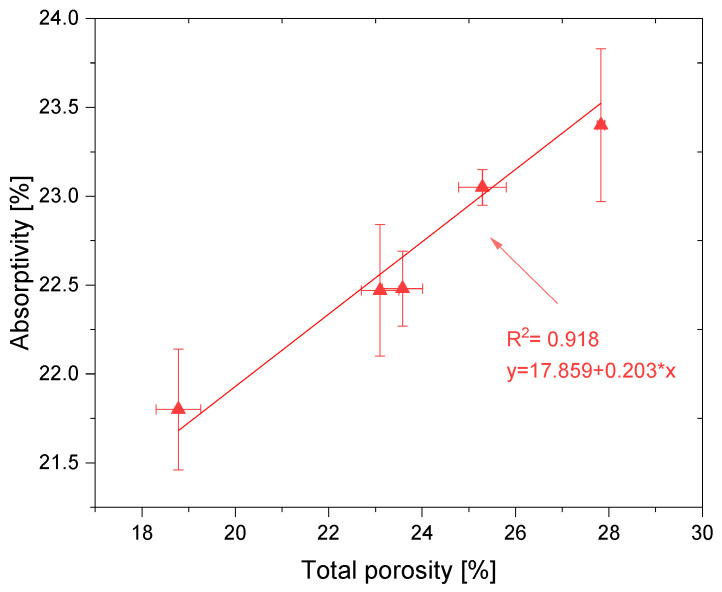
Correlation between the total porosity and water absorption.

Figure 6 shows the water absorption in a function of a porosity and a linear model of their relationship. Both parameters change with the amount of BP used. The linear trend is characterized by a good coefficient of determination R^2^ equal to 0.918. The total porosity of the mortars is the second most important parameter related to controlling the water absorption of the material: the higher the porosity, the higher the amount of water that can fill the pores. The water penetration behavior is corresponding to the pore distribution of concrete [57,58,59]. The BP addition gradually promoted its flow. These results suggest that the increase in porosity of hardened mortars is caused by the BP addition, and the differences in microstructure are due to its physical properties. BP particles increase the pore volume in the cured matrix [60,61]. The reduction in density leads to an obvious increase in porosity [62].

**Figure 7:**
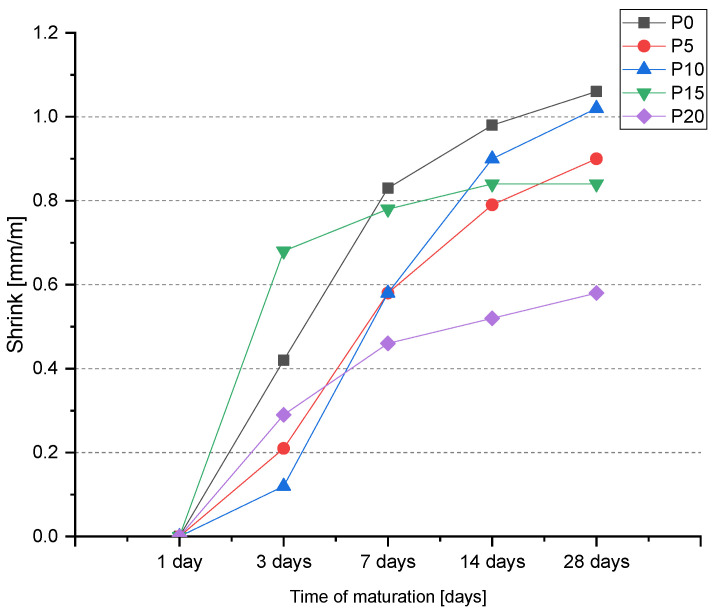
Linear shrinkage of the BP-modified cement mortars.

The effect of BP addition on linear shrinkage (Figure 7) is already visible after 3 days. P5 exhibited half the shrinkage of the reference sample. The smallest early shrinkage was recorded for P10 at 0.12 mm/m, which was 71.4% less than that of the P0 mortar. The P20 mortars also showed 69.1% less shrinkage after 3 days of curing. After 7 days of curing, the effect of BP is still visible. Mortars P5, P15, P20 reached lower linear shrinkage as well as lower shrinkage increment than the P0. The mortar with the highest BP content had the lowest shrinkage value. The addition of 20% reduced shrinkage by 44.6% relative to P0. Test results after 14 days of maturation confirm previous trends. Samples P5, P10, P15, and P20 achieved less shrinkage than P0. After 28 days of maturation, the last shrinkage measurement was performed. All series of BP-modified mortars showed less shrinkage than the reference sample. The lowest value of linear shrinkage was achieved by the mortar with the highest BP addition, and was 45.3% lower than the P0. The average decrease in shrinkage for all BP-modified mortars was 21.3%. The increase/decrease in shrinkage is mainly due to the significant evaporation of the batch water for the mortar tested. Taking into account that the shrinkage value decreased with BP addition, it means that in this case the kinetics of cement hydration reaction decreases with BP addition [38]. Similar relationships were observed by other researchers in their studies [51].

#### 3.3.2. Compressive Strength

**Figure 8:**
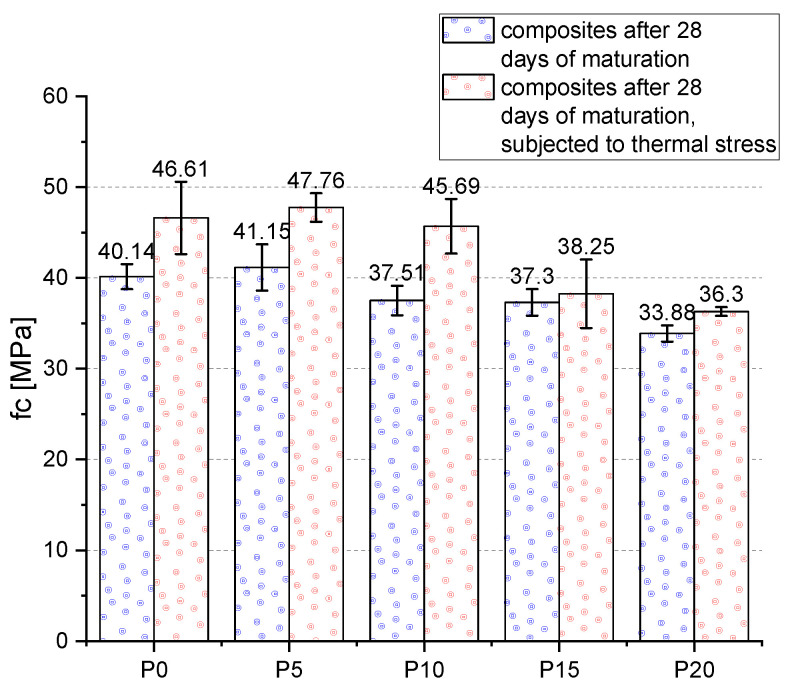
Compressive strength of mortars after 28 days of maturation.

The most favorable f_c_ results for mortars subjected to thermal loading were obtained for mortars with 5% wt. BP additive (47.76 MPa)—Figure 8. The highest increase in f_c_ in comparison to the samples that were not exposed to elevated temperature was achieved by the mortar without BP additive—23.4%. With the increase of BP content, the f_c_ increment successively decreased. It proves the negative influence of BP on the examined parameter. The short-term thermal loading caused an increase in mortar strength. The average increase in f_c_ for all the samples was 14.4%. Temperature loading leads to either a change in the calcium concentration in the mortar or a shift in the solubility equilibrium of portlandite which results in a large change in the properties of the C-S-H formation [63]. The increase in hydration products leads to an increase in its strength. This effect is due to the higher mobility of water molecules in the gas phase (compared to their mobility in liquids) for temperatures between 100 and 300 °C [64]. It should be noted that bound water starts to release at around 180 °C [65]. This observation is similar to the findings reported by Lim et al. [66] and Heikal et al. [67]. An increase in strength in the temperature range 105–200 °C can be explained by further hydration of unhydrated cement particles and/or an improvement of the local mechanical properties of C-S-H. This is the so-called “internal autoclaving” phenomena [68,69].

**Figure 9:**
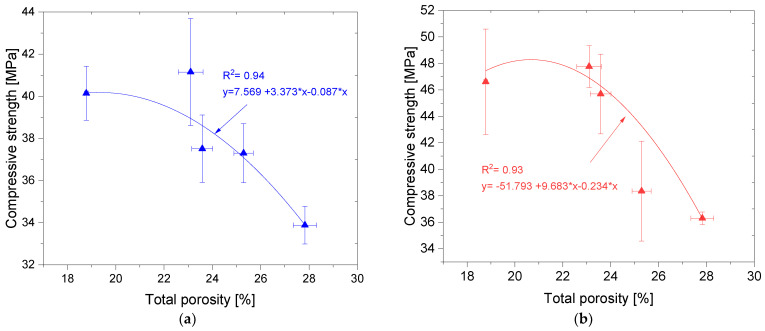
Relation between the total porosity and compressive strength: (**a**) reference samples, (**b**) samples subjected to the thermal stress.

Figure 9 shows the relationship between f_c_ and P_T_. The relationship between f_c_ and P_T_ is best described by a second-order polynomial function. The function has a very good coefficient of determination R_2_ of 0.94 (for standard specimens) and 0.93 (for specimens subjected to the thermal load). There is a proportional relationship between f_c_ and P_T_. The higher the porosity, the more the strength decreases [70]. It is worth noting that in the case of the mortars studied, a sudden decrease in f_c_ occurs when P_T_ increases above 23%. For lower porosity the mechanical characteristics are relatively constant.

#### 3.3.3. Flexural Strength

**Figure 10:**
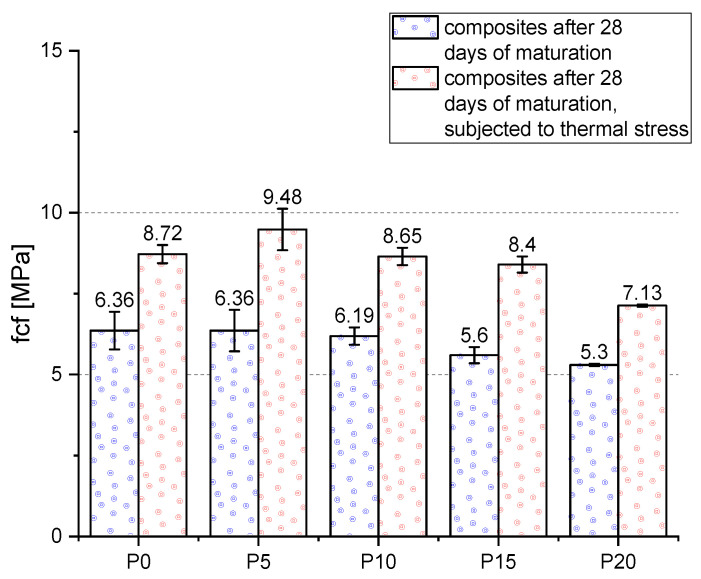
Flexural strength of the mortars after 28 days of maturation.

The highest f_cf_ value among the reference samples was obtained for the P0 and mortar with 5% wt. BP addition (6.36 MPa)—Figure 10. f_cf_ decreased above 5% wt. BP addition. The lowest strength was obtained for mortars with 20% wt. BP addition. The relationship between the amount of additive and f_cf_ mirrors the f_c_—the strength decreases above the 5% of the additive. Similar results were obtained by other researchers who observed an increase in f_cf_ for BP additive below 10% [28,38]. In addition, researchers have observed a significant increase in strength after 90 days of curing [71].

Similar to f_c_, the results were also improved after thermal loading. This parameter increases after subjecting the samples to thermal loading in the range of 100–200 °C. Under the influence of an elevated temperature, the chemical composition and physical structure of the cement paste changes significantly, the C-S-H phase is remodeled [72]. In addition, the thermal shock resistance of cement composites is influenced by such variables as modulus of elasticity, fracture energy, thermal conductivity or thermal expansion [73]. The best results among the mortars exposed to high temperatures were obtained by samples with 5% wt. BP addition (9.48 MPa). Then, the P0 (8.72 MPa), P10, P15, P20 (8.65 MPa, 8.40 MPa, and 7.13 MPa). The BP had a positive effect on the studied parameter. The f_cf_ increase was the highest for the samples with 15% wt. BP addition (strength increase of 50% compared to the samples unloaded with temperature). Then, the greatest increase was obtained for samples P5 (49% increase), P10 (40% increase), P0 (37% increase), P20 (34% increase). For P5, the increase in f_cf_ due to the adhesion of the cement matrix to the rough surface of the BP grains equivalent the effect of reducing the cement content. For higher BP contents, the decrease in binder content is the main factor responsible for the decrease in f_cf_ values.

**Figure 11:**
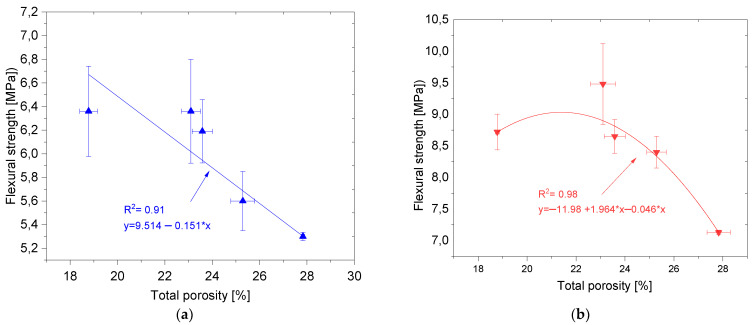
Relation between the total porosity and flexural strength: (**a**) reference samples, (**b**) samples subjected to the thermal stress.

Figure 11 also shows the relationship between f_cf_ and P_T_. The best fit to the empirical data is given by a linear function (for standard samples) and a second-degree polynomial for samples subjected to the thermal load. Both functions were characterized by very good coefficient of determination R^2^ equal to 0.91 (for standard samples) and 0.98 (for samples subjected to the thermal load). It is worth noting a similar relationship as in the case of f_c_-P_T_, i.e., a sudden decrease in f_cf_ with an increase in P_T_ above 23%.

#### 3.3.4. Ultrasonic Pulse Velocity

**Figure 12:**
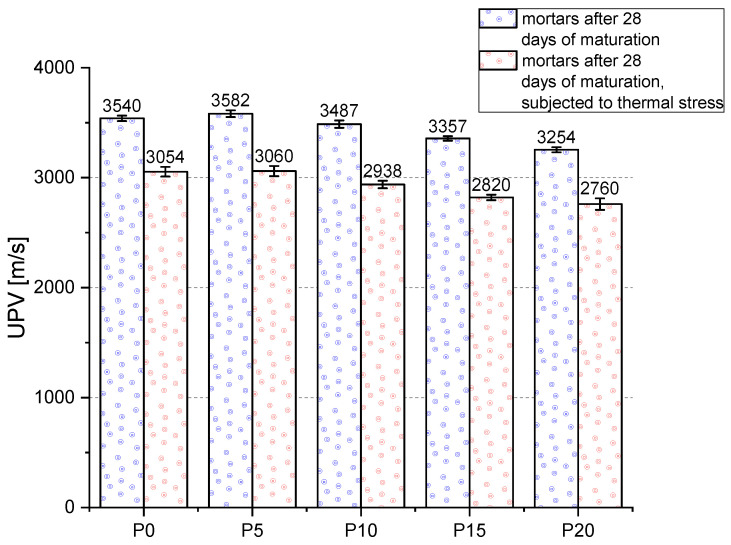
Results of the ultrasonic pulse velocity (UPV) measurements.

The variation of the UPV for reference mortars and after thermal load is shown in Figure 12. The highest UPV value for reference samples was obtained for P5 mortars, the result was 1.2% higher than for P0 mortars. With a higher amount of additive, the value of the index successively decreased. The lowest pulse velocity was obtained for P20 samples, which had the highest porosity. Similar studies were also conducted by other researchers [74,75,76]. The pulse velocity after thermal loading was lower compared to the reference samples. This is related to the water donation of the sample, remodeling of the C-S-H phase and increase in the porosity of the sample. The decrease in UPV with increasing BP content is related to the porous structure of BP grains. With higher BP content, the resultant porosity of the sample increases. The differences in UPV values between the series should be considered statistically significant because the scatter of results within a single series is usually very small.

#### 3.3.5. Scanning Electron Microscopy

**Figure 13:**
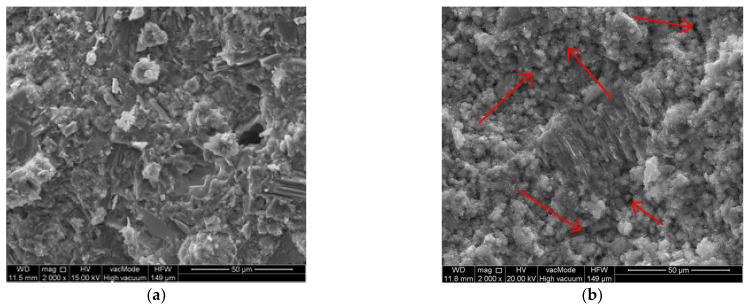
Scanning electron microscope (SEM) images of P0 mortars: (**a**) before being subjected to the thermal stress; (**b**) after being subjected to the thermal stress.

Figure 13 shows the P0 mortars before and after thermal loading. The matrix structure in the reference samples is compact and tight. The C-S-H phase takes the form of a compact gel with clusters of fibrous forms. After temperature exposure, the local porosity of the cement matrix increases due to the loss of free water. This is manifested by discontinuities in the structure of the C-S-H phase. In Figure 13b, the zones of an increased porosity (black spots) are indicated by arrows. The observations also confirmed the thermal stability (up to 250 °C) of the CSH phase, as it did not undergo a significant transformation in terms of morphology. It was also observed that there was a higher degree of cement overreaction for the samples subjected to the thermal load. This corresponds to the previously mentioned phenomenon of “internal autoclaving” [77].

#### 3.3.6. Prediction of Strength Properties of Mortars with BP

Observations made by other researchers indicate that, for concretes and cement mortars, the relationship between f_c_ and UPV is usually very close [78]. The problem arises when trying to predict the mechanical properties of samples thermally exposed to 250–400 °C, because on the one hand f_c_ increases in this temperature range due to the “internal autoclaving” process, and on the other hand the evaporating water causes an increase in porosity and a decrease in the UPV. Thus, the f_c_-UPV relationship is different when compared to cementitious composites not subjected to thermal loading [69]. To solve this problem, a supervised machine learning algorithm in the form of SVM (support vector machines) regression approach was implemented in this paper. This approach works very well for developing regression models for low-dimensional data sets [79].

**Figure 14:**
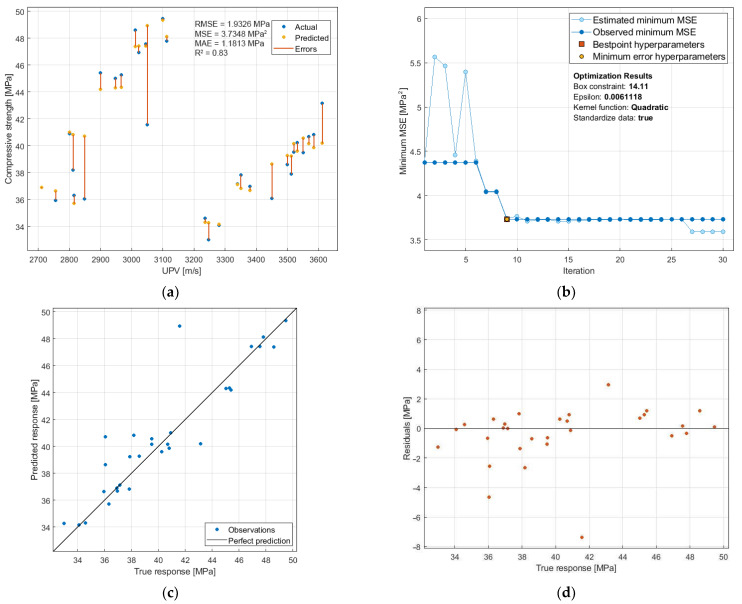
Support vector machine (SVM) prediction of the compressive strength: (**a**) f_c_-UPV relation with prediction errors; (**b**) optimization plot; (**c**) predicted-measured values relation; (**d**) distribution of residuals; RMSE—root mean square error; MSE—mean square error; MAE—mean absolute error; R^2^—coefficient of determination.

Figure 14 shows the results of SVM modeling for the f_c_-UPV relationship. The model was optimized in the criterion of the smallest MSE value, so that the prediction accuracy is as high as possible. Parameters optimized included the box constraint (0.001–1000), kernel scale (0.001–1000), epsilon (0.006–600), kernel function (Gaussian, linear, quadratic, cubic), and standardization of data (true, false). The Bayesian optimization with acquisition function in the form of expected improvement per second plus, with a maximum of 30 iterations was performed. Analyses were performed using Matlab software.

The f_c_-UPV relationship shown in Figure 14a indicates the formation of two groups of results. Those on the right correspond to the standard samples, those on the left to the samples after the thermal load. Such grouping of results makes it impossible to implement the traditional approach to the prediction problem, i.e., by using continuous functions, e.g., a linear or polynomial. In spite of the fact that the R^2^ value for the model does not indicate a very good (>0.90) but only a good (0.83) fit to the empirical values, the SVM approach used allowed us to obtain a model with a very good prediction level, since MAE reached a very low value of 1.1813 MPa, with f_c_ values in the range of 33–50 MPa. The most accurate model was obtained already in the 9th optimization step (Figure 14b), and further iterations did not improve the model accuracy. Analysis of the distribution of residuals (Figure 14d) indicates that the vast majority of errors are in the range of −1.6 MPa to 1.6 MPa.

**Figure 15:**
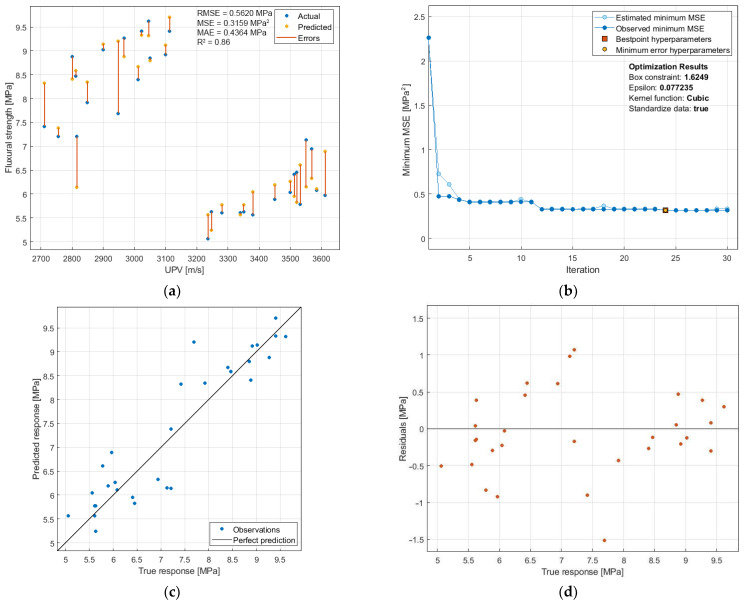
SVM prediction of the flexural strength: (**a**) f_cf_-UPV relation with prediction errors; (**b**) optimization plot; (**c**) predicted-measured values relation; (**d**) distribution of residuals.

The relationship between f_cf_ and UPV is also characterized by a two-group distribution of results (Figure 15a), depending on whether the samples were exposed to temperature or not. The developed SVM model and its optimization were performed under the same assumptions as for the f_c_-UPV relationship. In this case, a higher degree of model fit (R^2^ = 0.86) was obtained compared to the f_c_-UPV relationship. The MAE value is very low (0.4364 MPa), which, with f_cf_ results obtained in the 5–10 MPa range, indicates a prediction error of less than 9%. Considering that f_cf_ is not a leading mechanical characteristic for cement mortars with BP, the prediction accuracy should be considered high. The model with the highest accuracy was obtained with 24 iterations (Figure 15b), with cubic kernel function (with f_c_-UPV, the highest accuracy was obtained with quadratic kernel function). The analysis of the distribution of residuals (Figure 15c,d) shows their more uniform scatter compared to f_c_-UPV.

The application of the SVM regression approach allowed us to create accurate predictive models of f_c_ and f_cf_ based on UPV, which can be applied in practice. Their implementation is of particular importance for cement mortars modified with BP additive, the structure of which has been subjected to elevated temperature loading.

## 4. Conclusions

The present study investigated the physical and mechanical properties of mortars with BP under thermal shock conditions. The authors drew the following conclusions:

- The BP addition causes changes in the physical properties of mortars. Along with BP additive the D and ρ decreases. There is an increase in porosity and water absorption, and thus a decrease in tightness. Samples P0 were characterized by the highest tightness. This may indicate the lack of pozzolanic action of BP resulting in no increase of C-S-H phase in the hardened matrix.
- The increase in porosity of samples with BP addition resulted in a decrease in f_c_ and f_cf_ of mortars. The best results were obtained for samples with 5% wt. BP addition. Above this addition f_c_ decreased. The same relation was observed for f_cf_. The strength properties tested suggest that moderate BP addition up to 5% is the most beneficial.
- The f_c_ and f_cf_ for samples subjected to the thermal load increases in comparison to the reference samples (not exposed to the elevated temperature). The phenomenon of “internal autoclaving” occurs here, where on one hand the strength increases as a result of the C-S-H phase remodeling, and on the other hand the evaporating water causes an increase in porosity, which manifests itself, among other things, in a decrease in the UPV.
- The addition of BP positively influenced the linear shrinkage. The lowest value of linear shrinkage was achieved by the mortar with the highest BP addition, which was 45.3% lower than the classical mortar.
- The application of the SVM regression approach technique allowed us to create accurate f_c_ and f_cf_ predictive models based on UPV, which can be applied in practice.

According to the results, it is suggested that BP can be used as a cement replacement in the production of cement mortars, but the percentage of addition should be moderate and adjusted to the characteristics that mortars should have. The authors also plan to test mortars with BP addition at longer curing times to study the effect on subsequent strength characteristics.

## Figures and Tables

**Table 1:** Compositions of mortars [%].

	P0	P5	P10	P15	P20
Cement CEM I 42.5R	25	23.75	22.5	21.25	20
Quartz sand 0/2 mm	75	75	75	75	75
BP	0	1.25	2.5	3.75	5

**Table 2:** Physical properties of mortars.

	P0	P5	P10	P15	P20
Apparent density D [kg/m^3^]	1.94	1.93	1.93	1.91	1.91
Specific density ρ [kg/m^3^]	2.69	2.58	2.55	2.49	2.35
Tightness T [%]	81.22	74.71	76.90	76.42	72.17
Total porosity P_T_ [%]	18.78	23.10	23.58	25.29	27.83
Water absorption W_A_ [%]	21.80	22.47	22.48	23.05	23.40

## Data Availability

The data presented in this study are available on request from the corresponding author. The data are not publicly available as the data also forms part of an ongoing study.
